# Effect of mutation mechanisms on variant composition and distribution in *Caenorhabditis elegans*

**DOI:** 10.1371/journal.pcbi.1005369

**Published:** 2017-01-30

**Authors:** Ho-Yon Hwang, Jiou Wang

**Affiliations:** Department of Biochemistry and Molecular Biology, Bloomberg School of Public Health, Department of Neuroscience, School of Medicine, Johns Hopkins University, Baltimore, MD, United States of America; University of Texas at Austin, UNITED STATES

## Abstract

Genetic diversity is maintained by continuing generation and removal of variants. While examining over 800,000 DNA variants in wild isolates of *Caenorhabditis elegans*, we made a discovery that the proportions of variant types are not constant across the *C*. *elegans* genome. The variant proportion is defined as the fraction of a specific variant type (e.g. single nucleotide polymorphism (SNP) or indel) within a broader set of variants (e.g. all variants or all non-SNPs). The proportions of most variant types show a correlation with the recombination rate. These correlations can be explained as a result of a concerted action of two mutation mechanisms, which we named Morgan and Sanger mechanisms. The two proposed mechanisms act according to the distinct components of the recombination rate, specifically the genetic and physical distance. Regression analysis was used to explore the characteristics and contributions of the two mutation mechanisms. According to our model, ~20–40% of all mutations in *C*. *elegans* wild populations are derived from programmed meiotic double strand breaks, which precede chromosomal crossovers and thus may be the point of origin for the Morgan mechanism. A substantial part of the known correlation between the recombination rate and variant distribution appears to be caused by the mutations generated by the Morgan mechanism. Mathematically integrating the mutation model with background selection model gives a more complete depiction of how the variant landscape is shaped in *C*. *elegans*. Similar analysis should be possible in other species by examining the correlation between the recombination rate and variant landscape within the context of our mutation model.

## Author Summary

DNA variants in the world population of a species reflect the genetic diversity of the species. While examining variants in whole-genome sequenced wild isolates of the nematode worm *Caenorhabditis elegans*, we discovered apparent correlations between the recombination rate and the proportion of many variant types. To explain this correlation, we present a model of a concerted action of two groups of mutation mechanisms, which act according to different components of the recombination rate. This model can also explain how mutation mechanisms as a whole can affect the genomic landscape of the variant distribution. Using this mutation model, we systemically and holistically estimate the probabilities of generating specific mutation types by distinct groups of mutation mechanisms in *C*. *elegans*. Since this mutation model can be mathematically combined with existing natural selection models, such as background selection, a more comprehensive analysis of genetic diversity is now possible. We expect that the mathematical equations we present here can be used for refining computer simulations of evolution and coalescent modeling. Within the context of this mutation model, the correlation between the recombination rate and the proportion of variant types may serve as a useful new metric for analysis of genetic diversity in other species.

## Introduction

Genetic diversity is maintained by interplay between the continuing generation and removal of variants; variants are produced by mutation and removed by genetic drift or natural selection during evolution. The modes of generating mutation are biologically diverse, and variants accumulate throughout the life history of a species [1]. Natural selection can drive non-neutral variants to extinction or fixation. Neutral variants can be removed either by genetic drift [2], which is driven by simple chance, or by natural selection because of linkage or proximity to a non-neutral variant [3, 4]. The variants that are closely linked to a beneficial mutation may become concurrently fixed as a result of selective sweeps [3], and the variants that are closely linked to a deleterious mutation can become concurrently extinct as a result of background selection [4]. Thus genetic diversity is shaped by both mutation and natural selection.

In many species including *Caenorhabditis elegans*, higher frequency of variants is observed in the regions of high recombination [5–7]. Natural selection, notably selective sweep and background selection, is the favored explanation for the existence of this pattern [8], but other proposed explanations include elevated rates of mutation associated with higher recombination, sequestration of important DNA or genes in the regions of low recombination, and sequencing analysis error [9, 10]. The recombination-based mutation mechanism was arguably ruled out in *Drosophila melanogaster* early on [5] but was raised as a possible explanation for the variant distribution in humans [11, 12]. In *C*. *elegans*, background selection in combination with selective sweep were shown to shape the variant distribution [7, 13, 14], but a study of codon usage suggested that elevated rates of mutation exist in regions of high recombination [15]. The analysis of *C*. *elegans* mutation accumulation (MA) strains does not show a correlation between the recombination rate and the accumulation of mutations and thus strongly argues against a substantial role of mutation [16, 17], but it is possible that culturing condition in the laboratory leads to mutation rates that do not reflect the mutation rates in the wild environment. Thus in shaping the variant distribution, natural selection is generally agreed as an important factor while mutation is thought to play a lesser role in *C*. *elegans* [7, 13] and perhaps an insignificant role in many species [18–20].

In the present study, we performed a more complete examination of genetic diversity by a previously untried analysis of the composition of variants (e.g. the proportion of specific variant types), which complements the standard analysis of the distribution of variants (i.e. variant frequency and density). Here, we use the term proportion for a single specific variant type and the term composition for proportions of all variant types. Over 800,000 homozygous variants present in 40 wild isolates of *C*. *elegans*, which had been whole-genome sequenced concurrently [10], form the basis of our analysis. First, we demonstrated a strong correlation between the recombination rate and the proportion of many specific variant types. This new correlation is as good as the known correlation between the recombination rate and variant distribution. This new correlation demands an explanation and provides a new avenue for studying genetic diversity. Most other genomic features, such as GC content, expression level, and essential gene exon density, show weaker correlation than the recombination rate whereas exon and repetitive sequence densities show a similar level of correlation as the recombination rate. The correlation between the recombination rate and variant composition remain strong using only the variants that do not affect exons or only the variants that affect the DNA outside repetitive sequences. We discuss natural selection and mutation as possible actors responsible for the correlation. To explain the correlation, we present a mutation model and associated mathematical equations, which are based on the premise of a combined action of mutation mechanisms with distinct basis of mutation generation probabilities. Using regression analysis, we estimated the contributions of different mutation mechanisms and their properties. The mutation model also can be used to re-examine the correlation between the recombination rate and variant distribution. Furthermore, the mutation model can be integrated with background selection model to depict a more complete history of the landscape of variants in *C*. *elegans*. In addition to explaining the correlation between the recombination rate and variant composition, our analysis suggests a greater importance of mutation in shaping the variant distribution in *C*. *elegans* than previously thought.

## Results and Discussion

### Variant composition is correlated with the recombination rate

**Fig 1:**
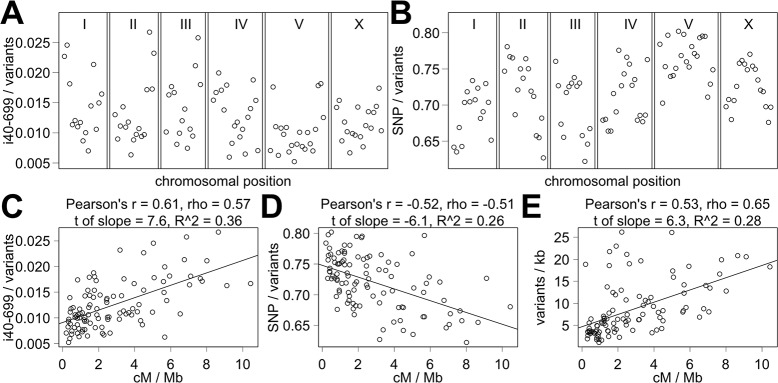
The landscape of variants by chromosomal position and recombination rate. (A-D) The proportions of i40-699 out of all variants (A, C) and SNPs out of all variants (B, D) are shown by chromosomal position (A, B) and relative to the recombination rate (C, D). (E) The distribution of all variants relative to the recombination rate. Black lines indicate the line of best fit by linear regression.

We discovered a striking pattern while examining the composition of variants such as indels of size between 40 and 699 base pairs (i40-699). The proportion of i40-699 out of all variants is higher near autosomal ends and lower in autosomal centers (Fig 1A). For every genomic interval, the proportion of i40-699 out of all variants is calculated by dividing the number of i40-699 by the number of all variants. All variants include all SNPs, all indels, and all other complex variants. For example, a genomic interval with 70 SNPs, 30 indels including a single i40-699, and no other complex variants has 1% i40-699/variants by this metric of variant type proportions. By polymerase chain reaction (PCR) assay, we have positively verified at least 121 out of 124 (97.5%) i40-699 in CB4856, which suggests a high quality of variant calling for the indels of this size range. The pattern of higher proportion of i40-699 out of all variants in autosomal ends is reminiscent of the pattern of variant distribution reported in prior publications [7, 10, 21, 22]. The underlying reason for the pattern of the variant distribution was attributed to the associated recombination rate, which is higher in autosomal ends, and thus we tested the correlation between the recombination rate and the proportion of i40-699 out of all variants.

The strength of correlation can be examined by a number of correlation tests including the methods of Pearson [23], Spearman [24], and Kendall [25] as well as by simple linear regression. The possible range of values for Pearson's r, Spearman's rho, Kendall's tau is between 1 and -1 with a larger absolute value indicating a stronger correlation. Using linear regression, useful indicators include the t value associated with the slope and the R^2^ value. Correlation tests show a strong positive correlation between the recombination rate and the proportion of i40-699 out of all variants (Fig 1C, Pearson's r = 0.61, Spearman's rho = 0.57, Kendall's tau = 0.4). By linear regression, the t value of 7.6 associated with the slope indicates that the slope value is 7.6-fold greater than the standard error (*p* < 0.001, R^2^ = 0.36). A positive correlation means that the proportions of i40-699 out of all variants are higher in the regions of high recombination. A strong correlation raises the possibility of recombination rate being a causative factor in establishing different proportions of i40-699 out of all variants across the genome.

**Fig 2:**
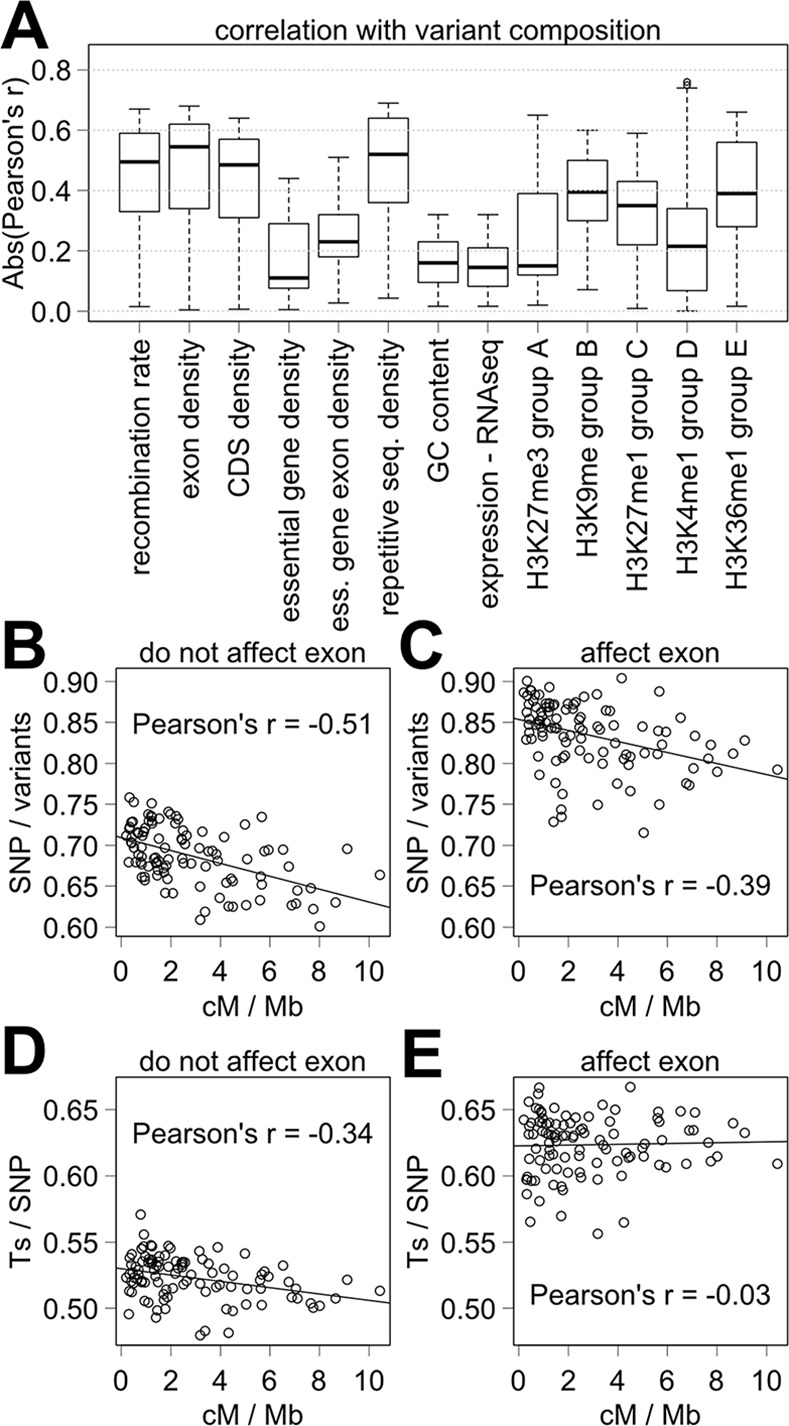
Correlation between variant composition and recombination rate and other genomic features. (A) Box plot of Pearson's r absolute values for the correlation between variant composition and various genomic features. (B-E) Proportions of SNPs out of all variants (B, C) and of Ts out of all SNPs (D, E) relative to recombination rate for the variants that do not affect exons (B, D) and for the variants that affect exons (C, E).

For a more thorough analysis of variant composition, we examined the proportions of 30 variant types out of all variants, the proportions of 26 non-SNP subtypes out of all non-SNPs, and the proportion of transitions (Ts) and transversions (Tv) out of all SNPs. Indel variant types range from indels of single base pair change (i1) to indels of 100 to 4999 base pair change (i100-4999). We define non-SNP as variant types encompassing indels and substitutions of two or more adjacent base pairs as well as combination of these mutation types, which include variants without any change in the number of base pairs (i0) but is not a SNP. The proportions of SNPs out of all variants and transitions (Ts) out of SNPs showed strong negative correlation with the recombination rate with lower proportion in the regions of high recombination (Fig 1D, S1 Table). A negative correlation for SNPs out of all variants means that SNPs constitute a smaller proportion of all variants in the regions of high recombination, which coincide with autosomal centers (Fig 1B). A negative correlation does not mean that there are fewer SNPs in the regions of high recombination, and in fact there are more SNPs in the regions of high recombination. Most variant type proportions show a strong positive correlation with the recombination rate (S1 Table). The range of Pearson's r using the absolute value is from 0.015 to 0.67 with 25% quartile of 0.33, median of 0.50, 75% quartile of 0.59, and mean of 0.45 (Fig 2A). The weakest correlations are associated with i0, i1, i4 and i5 as indicated by the weak Pearson's r values for i0/non-SNPs (-0.015), i1/all variants (0.17), i4/non-SNPs (-0.16), and i5-9/non-SNPs (-0.07). Similar conclusions can be made using Spearman's rho and Kendall's tau (S1 Table). Thus, strong correlation with the recombination rate exists for the proportion of most but not all variant types.

As a comparison, we examined the well-known correlation between the recombination rate and the distribution of variants using the same analysis. Correlation tests show strong positive correlation for all variants (Pearson's r = 0.53, Spearman's rho = 0.65, Kendall's tau = 0.46), and linear regression analysis shows a t of slope of 6.3 and R^2^ of 0.28 (Fig 1E). The correlation is positive for all variant types, but the correlation is somewhat weaker for SNP (Pearson's r = 0.46), Ts (Pearson's r = 0.42), Tv (Pearson's r = 0.5), and especially i0 (Pearson's r = 0.25). All other indels including i40-699 show a strong positive correlation (Pearson's r = 0.71 for i40-699, S2 Table). Using Spearman's rho and Kendall's tau, the trend is similar but with smaller differences in the strength of correlation among different variant types (S2 Table). Together, the strength of the newly observed correlation between the recombination rate and variant composition is similar to that between the recombination rate and variant distribution.

### The correlation is weaker in the X chromosome

The correlation between the recombination rate and variant composition is similar among autosomes, but the correlation is often weaker for the X chromosome (S2 Fig). Interestingly, the established correlation between the variant distribution and the recombination rate is also weaker for the X chromosome (S2E Fig). This suggests that if recombination rate is indeed a contributing factor in the distribution and composition of variants, the effect is smaller in the X chromosome.

### The correlation between variant composition and recombination rate compared to correlations between variant composition and other genomic features

To further test the relevance of the correlation between the recombination rate and variant composition, we also examined the correlation between variant composition and several other genomic features. Other genomic features examined are exon density, coding DNA sequence (CDS) density, essential gene density using either the gene length or exon length, repetitive sequence density using inverted and tandem repeats, GC content, expression level, and five different measures of chromatin state. Some of these factors, including exon density and repeat sequence density, are correlated with the recombination rate in *C*. *elegans* [26, 27]. Chromatin states in *C*. *elegans* have been placed into five groups [28], and some of these chromatin states (e.g. methylation state of histone H3K4me1 as measured by ChIP-seq) are known to be correlated with other genomic features, such as expression level or repeat sequence density [28]. Details of the results including all *p* values are shown in S3 and S4 Tables, and highlights are summarized in the next two paragraphs.

Using 58 definitions of the proportion of variant types, the correlations with the recombination rate show Pearson's r median absolute value of 0.5 (Fig 2A, S1 Table). Examining the correlations between the proportion of variant types with other genomic features instead, the correlations are essentially of same strength with the repetitive sequence density (Pearson's r median absolute value = 0.52), with the exon density (Pearson's r median absolute value = 0.54), and with CDS density (Pearson's r median absolute value = 0.48) (S3 Table). The correlations are weaker with all other factors. The Pearson's r median absolute values between 0.3 and 0.4 are observed for H3K36me1, H3K9me1, and H3K27me1 histone methylation states. The Pearson's r median absolute value is less than 0.3 for all other factors including essential gene exon density. Comparable results were obtained using Kendall's tau, and Spearman's rho suggests a somewhat stronger correlation with exon and repetitive sequence densities (S1 and S3 Tables). In summary, the correlation between the recombination rate and variant composition is among the strongest with the correlations between variant composition and exon or repetitive sequence densities showing a similar level of correlation.

The distribution of variants was also examined as a comparison. Using the distribution of 31 different types of variants, the correlations with the recombination rate show a Pearson's r median value of 0.70 (S2 Table). Examining the correlations between the distribution of variants and other genomic features instead, the correlations are slightly stronger with the repetitive sequence density (Pearson's r median = 0.75) and weaker with the exon density (Pearson's r median = -0.57). By Pearson's r, the correlations are weaker with all other factors (S4 Table). Comparable results were obtained using Spearman's rho (S2 and S4 Tables). Using Kendall's tau, the strongest correlation was observed with the recombination rate (tau median = 0.72) followed by repetitive sequence density (tau median = 0.57). Taken together, the relative strength of the correlation between the recombination rate and variant composition compared to other genomic features is arguably as strong as the relative strength of the correlation between the recombination rate and variant distribution.

### The correlation of variant composition is strong outside exons or repetitive sequences

We asked whether exon density or repetitive element density, which are correlated with the recombination rate, might be responsible for the existing landscape of variant composition rather than the recombination rate. First, we examined the correlation using only the variants that affect exons as opposed to using the variants that do not affect exons. By Pearson's r, similarly strong correlations are observed using all variants (median absolute value = 0.5) and using only the variants that do not affect exons (median absolute value = 0.53) (S5 Table). On the other hand, considerably weaker correlations are observed using only the variants that affect exons (Pearson's r median absolute value = 0.12) (S6 Table). Similar conclusions can be made using Spearman's rho and Kendall's tau values. Thus, the variants that affect exons are a minor actor in shaping the correlation between the recombination rate and variant composition.

As for repetitive sequence density, we examined the correlation using only the variants that affect DNA outside repetitive sequences. This was done because examining the correlation by separating variants into those that affect and those that do not affect repetitive sequences was less satisfactory (S7 and S8 Tables). Using the variants that affect DNA outside repetitive sequences, the correlations are strong (Pearson's r median absolute value = 0.49) (S9 Table) unlike when using only the variants that are located inside repetitive sequences (Pearson's r median absolute value = 0.11) (S10 Table). Similar conclusions can be made using Spearman's rho and Kendall's tau values. For highly repetitive sequences, sequencing analysis and variant calling may be problematic, and perhaps different variant types are affected to a different level depending on the degree of repetition. However, we cannot discount the possibility of the real absence of correlation between the recombination rate and variant composition in repetitive sequences. In any case, repetitive sequences have only a minor effect in the observed correlation between the recombination rate and variant composition.

### Natural selection is not the main actor behind the correlation between the recombination rate and variant composition

Here, we discuss forces that can affect variant composition, and we will consider natural selection first. Natural selection, which acts by the fitness of phenotype, can be divided into the direct natural selection of non-neutral variants and the collateral removal of neutral variants, which depends on the linkage or the recombination rate between the selected non-neutral variant and nearby neutral variants. In the latter case, linkage-based selection models, such as selective sweep [3] and background selection [4], has been used to explain the correlation between the recombination rate and variant distribution in light of their effect in removing more neutral variants in the regions of low recombination. These linkage-based models predict an aspect of the Hill-Robertson effect [29, 30]. However, the Hill-Robertson effect, selective sweep, and background selection do not affect variant composition because linked neutral variants are removed regardless of their type (e.g. SNP and indel). In other words, linkage-based selection models cannot explain the correlation between the recombination rate and variant composition.

On the other hand, direct natural selection of non-neutral variants should affect variant composition. Here, exon density is a likely modifier. Notably, indels are more likely to be deleterious than SNPs in protein coding sequence [31], and transversions are more likely to cause a non-conservative amino acid change because of the nature of the triplet code and wobble position. Indeed, the proportion of SNPs out of all variants is quite different among the variants that affect exons (84%) as opposed to the variants that do not affect exons (72%) (Fig 2B and 2C, S11 Table). Likewise, transitions (Ts) are 64% of SNPs that affect exons and 54% of SNPs that do not affect exons (Fig 2D and 2E). Using 58 physical-distance forward (pf) intervals, there is a clear difference (two sample t-test, *p* < 0.001) in the proportions between these variant populations. The difference is even greater if the variants that affect CDS are examined instead (SNPs = 89% of all variants, Ts = 66% of SNPs). In comparison, the variant composition is similar for the variants that affect introns but not exons and for the variants that affect neither coding sequences nor introns (S3 Fig). Thus, direct natural selection appears to have a stronger effect on the variant composition in exons, for example, by preferentially removing indels over SNPs and Tv over Ts.

We asked whether direct selection or exon density, which happens to be correlated to the recombination rate, is responsible for the correlation between the recombination rate and variant composition. A simple model would predict a uniform variant composition in exons and a different uniform variant composition outside exons. Such a model by itself does not predict a correlation between the recombination rate and variant composition for the population of variants that affect exons as well as for the population of variants that do not affect exons. However, strong correlation exists between the recombination rate and variant composition for the variants that do not affect exons (S5 Table). Thus the action of direct natural selection simply according to the exon density cannot explain the correlation between the recombination rate and variant composition.

Even in exons and CDS, where natural selection is expected to be a stronger force, mutation and other factors likely act in shaping the variant composition. Larger indels are fewer in number than smaller indels. For example, the number of indels of 2 base pairs or i2 is ~2.7-fold that of i3, and the number of i3 is ~1.6-fold that of i4 (S11 Table). However, an i3 in many cases should be less deleterious than an i1, i2, or i4 given the structure of the triplet code in RNA translation. Examining only the variants that affect CDS, the number of i3 (n = 1072) is greater than the number of i2 (n = 508) but smaller than the number of i1 (n = 1583) (S11 Table). Thus, natural selection is not the sole factor responsible for the presence of fewer larger indels. For example, mutation in a cell may be more prone to generate a SNP rather than i1 and more prone to generate an i1 than i2 or larger indels. Coordinated defense of a cell or an organism against mutation, such as DNA repair and apoptosis, may also contribute to the relative rarity of larger indels. Direct natural selection is likely a smaller factor in determining variant composition outside exons, and conversely mutation should be of greater importance outside exons.

For direct natural selection to be responsible for the correlation between the recombination rate and variant composition, selection must act either according to the recombination rate itself or according to another genomic feature, which happens to be correlated to the recombination rate. Repetitive sequences are correlated with the recombination rate, but repetitive sequence density is unlikely to be the relevant factor because strong correlation exists between the recombination rate and variant composition for the variants that affect DNA outside repetitive sequences (S9 Table). Genomic features such as CDS, exons of essential genes, highly conserved domains, and regulatory elements all may be affected differently by direct selection. However, we think that all of these genomic features are unlikely to explain the correlation between the recombination rate and variant composition. Alone by natural selection, we are unable to conceptualize how disparity in the variation composition can be generated in a genome according to the recombination rate, although we cannot definitively eliminate the existence of such a mechanism. Later in this manuscript, we will discuss how natural selection may modify a pre-existing correlation between the variant composition and recombination rate. In brief, natural selection affects variant composition except probably not de novo by the recombination rate.

### Mutation can affect variant composition and distribution by recombination rate

Mutation is a good candidate for the causative factor that is responsible for the correlation between the recombination rate and variant composition. However, we need a more comprehensive model than simply stating that high recombination rate causes high mutation rate. For a more comprehensive model, we classified all mutation mechanisms by their relationship to the recombination rate. The recombination rate of a genomic interval is determined by the genetic distance and physical distance of the interval. We suggest that all mutation mechanisms can be classified into two groups, one dependent on the relative genetic distance and the other dependent on the relative physical distance. Two general assumptions are that relative contributions of the mutation mechanisms are constant and that there are fixed probabilities for the generation of different mutation types. This mutation model is discussed in depth below.

First, consider the mutation mechanisms that act according to the physical distance. Such a mutation mechanisms would generate three times as many variants in an interval of 3 megabase (Mb) compared to an interval of 1 Mb. A key underlying assumption is that the probability of mutation being generated is equal from one nucleotide to the next nucleotide. For example as a chromosome is replicated by a DNA polymerase, one nucleotide is as likely to become a site of mutation as the next nucleotide. The probability of mutation being generated at each base pair position during replication is assumed to be a constant. We named this group of mutation mechanisms as the Sanger mechanism for the critical role that the Sanger sequencing method played in determining the physical distance of DNA. We note that some chemical mutagens and irradiation may affect some nucleotide more than others, and homopolymers may be more liable to slippage resulting in a loss or a gain of base pairs. Such caveats aside, the number of variants generated by the Sanger mechanism for a genomic interval of a given physical length is defined as follows:

#variantsbySangermechanism=Sangercoefficient(S)*physicaldistance(n)

Next, consider the mutation mechanisms that act according to the genetic distance. Such mechanisms would generate twice as many variants in an interval of 2 centimorgans (cM) compared to an interval of 1 cM. Using the strictest definition, the probability of mutation generation here depends on the probability of a base pair position becoming a site of meiotic chromosomal crossover. We call this group of mutation mechanisms as the Morgan mechanism. Unequal crossover of chromosomes leading to mutation has been known from the early days of modern biology [32], and direct evidence for mutagenic effect of recombination was shown recently [33]. We hypothesize that the Morgan mechanism is not limited to mutations arising directly from errors in chromosomal crossovers. Specifically, chromosomal crossover is known to be preceded by programmed DNA double-strand breaks, which is catalyzed by Spo11 topoisomerase-like protein [34]. Meiotic double-strand breaks can be repaired through many different pathways with or without a chromosomal crossover [35]. Hotspots for double-strand breaks and the frequency of these hotspots may determine the recombination rate. Thus, a suitable definition of the Morgan mechanism may encompass mutations that are generated as a consequence of programmed DNA double-strand break that may lead to recombination. By this interpretation, the probability of meiotic DNA double-strand break is the primary contributor to a constant that we call the Morgan coefficient. The Morgan coefficient and the genetic distance of a given interval have the following relationship with the number of mutations generated by the Morgan mechanism:

#variantsbyMorganmechanism=Morgancoefficient(M)*geneticdistance(g)

To formulate a mathematical model that combines the effect of the Morgan and Sanger mechanisms, the contribution of the two mechanisms needs to be normalized to the same time scale. Therefore, both Morgan and Sanger coefficients should reflect the number of potential mutation generation events in a single generation. For example, the probability of mutation generation by the Morgan mechanism should be modified by ten if there are ten programmed meiotic DNA double-strand breaks in the life cycle of an organism starting from a fertilized egg to the next fertilized egg. Replication of a 100 Mb *C*. *elegans* genome would involve 100,000,000,000 base pairs with a probability of mutation at each base pair, and the sum of the probabilities should be multiplied by the average number of cell cycles involved going from one generation to the next. The number of variants generated by Morgan and Sanger mechanism in a single generation is defined as follows:

#ofvariantsgeneratedin1generation=(M*g+S*n)*1generation

There is a fixed ratio that describes the relative relationship between the Morgan and Sanger coefficients. We call this the R coefficient:

Rcoefficient=S/M(fixedratio)

Relative contributions of the Morgan and Sanger mechanisms can be written as shown below using the R coefficient and without the S coefficient:

$$\#\;\mathrm{of}\;\mathrm{variants}\;v_{o}=\;\left(r\;+\;R\right)\;*\;n\;*\;M\;*\;d$$

See S1 Text for the algebra used to derive the equation above. Here, r is the recombination rate, which is the genetic distance (g) divided by the physical distance (n). The R coefficient has the same units as the recombination rate. The factor d for divergence is the number of generations. The contribution of variant generation by the Morgan mechanism is r * n * M * d, and the contribution by the Sanger mechanism is R * n * M * d. We used uppercase letters for constants and lowercase letters for variables. We expect that a well-known population genetics term of mutation rate μ corresponds to the sum of (r + R) * n * M where r and n include all r and n values for all genomic intervals. A smaller R coefficient means that more contribution by Morgan mechanism and consequentially a higher density of variants in the intervals of high recombination rate. In brief, this equation can be used to examine the correlation between the recombination rate and variant distribution.

To explain the correlation between the recombination rate and variant composition, we need to discuss the probability of generating a specific variant type. The key assumption here is that there is a fixed probability of a mutation generated being a specific mutation type for each mutation mechanism. Thus, we expect that Morgan and Sanger mechanisms would have different probabilities of generating a specific mutation type. The coefficient F_M_ defines the probability of generating a specific variant type by a Morgan mechanism mutation generation. Likewise, F_S_ defines the probability of generating a specific variant type from a Sanger mechanism mutation generation. An F_M_ of 1 or 0 for SNP means that the Morgan mechanism generates SNP with a probability of 100% or 0%, respectively. The proportion of a specific variant type in an interval is:

$$\mathrm{proportion}\;\mathrm{out}\;\mathrm{of}\;\mathrm{all}\;\mathrm{variants}\;f_{o}=\;(r\;*\;F_{M}+\;R\;*\;F_{S})\;/\;\left(r\;+\;R\right).$$

For each interval, the numerator (r * F_M_ + R * F_S_) may correspond to the number of a specific variant type, and the denominator (r + R) may correspond to the number of all variants in the interval. The same equation can be used with denominator being something other than all variants (e.g. SNPs or non-SNPs, see S1 Text). For the proportion of a specific variant type, a larger F_M_ value than the F_S_ value should lead to a positive correlation between the recombination rate and the proportion. On the other hand, a smaller F_M_ value than the F_S_ value should lead to a negative correlation, and equal values of F_M_ and F_S_ should lead to a zero correlation.

While we started the mutation model without considering non-mutation factors, it may be worth discussing what else may affect the values of R and F coefficients. In our opinion, the values of both R and F coefficients can be affected by DNA repair and apoptosis, which may affect Morgan and Sanger mechanisms differently. Germline apoptosis in *C*. *elegans* is stochastic and is differently activated than somatic apoptosis [36], and DNA damage-induced apoptosis involves a conserved genomic integrity checkpoint pathway [37]. It is possible that generation of a larger indel may be more likely to trigger apoptosis in a germ cell even if there is no selective advantage for a specific small indel over a specific large indel. It is also conceivable that the rate of triggering apoptosis in response to DNA damage is different for meiosis and mitosis. Given these scenarios, incorporating their effect on the R and F coefficients into the mutation model may be useful for DNA repair and apoptosis. Furthermore, natural selection can act in a similar manner to change the variant landscape, which we will discuss in more detail later (see the last section of Discussion). In brief, our model invoking mutation and biological mechanisms that directly respond to the mutation can explain the correlation between the recombination rate and variant distribution as well as the correlation between the recombination rate and variant composition.

### R coefficient estimates from variant composition analysis suggest that Morgan mutation mechanism is important

Using the equation for the proportion of specific variant type, it should be possible to estimate the F_M_, F_S_ and R coefficients given the recombination rate of each genomic interval and the corresponding proportion of a specific variant type in the interval. The values that best fit the observation is determined by using least squares (LS) regression analysis [38, 39]. A simple analysis would use the recombination rate of each interval as defined, for example by WormBase [40], and the corresponding proportion of each specific variant type using all variants. However, such a simple analysis ignores the stronger effect direct natural selection has on exons. To avoid a potentially large complicating effect of direct natural selection, the main analysis was done using only the variants that do not affect exons.

A potentially complicating factor in analyzing only the variants that do not affect exons is that a substantial part of the physical length of the genome is occupied by exons. Notably, 17.2% of all variants affect CDS, and 24.1% of all variants affect exons (S11 Table) whereas CDS and exons occupy 25.1% and 31.4%, respectively, of *C*. *elegans* chromosomes (S12 Table). Given that a large part of each genomic interval is being removed from the analysis, it may be worth asking whether the recombination rate of each genomic interval should be recalculated. Such a recalculation is possible only to a limited degree because genetic positions have been determined at the level of genes only rather than at the level of exons. For simplicity, we chose not to recalculate the recombination rate of each interval using a simplistic (although not exactly correct) assumption that exon density is even within each interval. With an even distribution of exons, we can assume that the genetic distance and the physical distance is removed in an equal and proportionate manner when exons are excluded from the analysis.

To obtain meaningful estimates using the standard pf interval boundary definition alone, we used aggregated data from 37 wild isolates for our main analysis. Only 37 of the 40 individual isolates were used because a trio (MY2, MY14 and JU1171) and a pair of the isolates (ED3057 and ED3072) are nearly identical throughout the entire genome, as also noticed by others [41]; ED3072, JU1171, and MY14 were removed from the analysis. Only genomic intervals with minimum of 300 variants were used.

**Fig 3:**
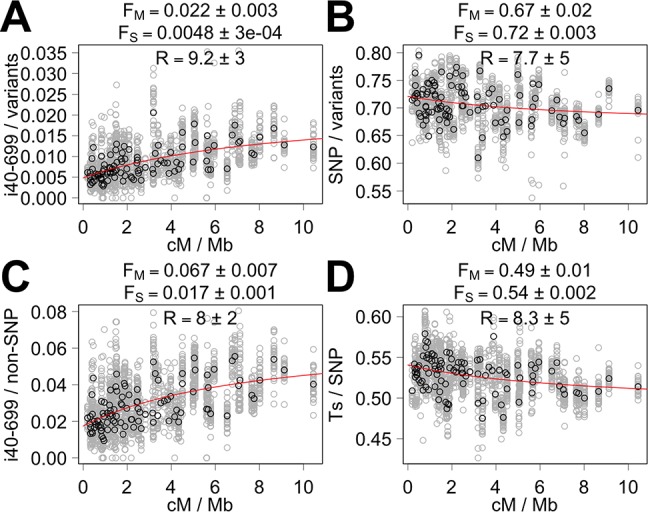
Fitting the correlation between the recombination rate and variant composition using the mutation model. The proportion of i40-699 out of all variants (A), SNPs out of all variants (B), i40-699 out of non-SNPs (C), and transitions (Ts) out of SNPs (D) for all segments with >300 variants outside exons are shown relative to the recombination rate (gray circles). Black circles indicate the mean proportions for different intervals. Red line indicates the line of best fit using all segments according to least squares regression using the mutation model.

First, we show the analysis of a small set of the proportion of variant types with easily interpretable results. Using the proportions of SNPs out of all variants for the aggregation of individual isolates, F_M_ and F_S_ are 0.67±0.02 (standard error of the estimate) and 0.72±0.00, respectively (Fig 3B). This means that 67% of the mutations generated by the Morgan mechanism are SNPs whereas 72% of the mutations generated by the Sanger mechanism are SNPs. Thus, both mutation mechanisms produce primarily SNPs with a somewhat higher probability for the Sanger mechanism. Examination of yeast *RAD52* recombinational repair mutants revealed a higher frequency of point mutations than other mutation types [42], and thus the estimation that Morgan mechanism generates SNPs predominantly is not surprising. Using the proportions of i40-699 out of all variants, the estimated probabilities of generating i40-699 are 2.2±0.3% and 0.5±0.0% for the Morgan and Sanger mechanisms, respectively (Fig 3A), which suggest that the Morgan mechanism is >4-fold more likely to generate an i40-699 than the Sanger mechanism. Meanwhile, the estimates of the R coefficient based on i40-699 and SNPs out of all variants are 9±3 and 8±5, respectively. Using i40-699 out of non-SNPs and Ts out of SNPs, the R coefficient estimates are 8±2 and 8±5, respectively (Fig 3C and 3D). An R coefficient of 8 suggests that 26% of all the variants in *C*. *elegans* are generated by the Morgan mechanism in the wild environment.

For a more complete analysis, we examined the correlation with the recombination rate using 58 definitions of the proportion of variant types. A wide range of the estimates of the R coefficient was obtained with a wide range of accompanying t and *p* values in our main analysis (S13 Table). We also present the estimates derived from LS regression using only the variants that do not affect CDS as well as using all variants (S14 and S15 Tables). We also present the estimates derived using a highly processed variant set, which involved combining overlapping variants and censoring of overshadowed variants (S16 Table, see Methods). In addition, we present the estimates derived from the whole variant data, which produce estimates with poorer t and *p* values, rather than aggregated data from 37 wild isolates (S17 Table). Here, better estimates need to be sorted from questionable estimates.

**Fig 4:**
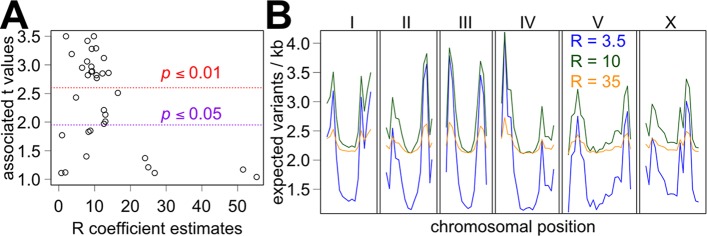
The best estimates of the R coefficient and the implications of R coefficient in the generation of skewed variant distribution. (A) The R coefficient estimates with the highest confidence as indicated by the associated t values of > 1. Possible cutoffs using *p* < 0.05 (purple) and *p* < 0.01 (red) are indicated by colored dotted lines. (B) Expected variant distribution by chromosomal pattern assuming an R coefficient value of 3.5 (blue), 10 (green), and 35 (orange). The presumed M * d values are 300, 205, and 60, respectively, for R coefficient values of 6, 10, and 60.

The quality of the R and F coefficient estimates can be assessed by examining the associated t and *p* values. For example, an R coefficient value below zero is biologically irrelevant, and thus any estimate that suggests the possibility of the true R coefficient value below zero can be considered weak. Such weak estimates of the R coefficient can be censored by using the t value associated with the estimate of R. Using a cutoff of t > 1, the R coefficient estimates range from 0.7 to 55.3 with a median value of 9.6 (Fig 4A). Thirty-four of 56 (61%) definitions of the proportion of variant types pass the t > 1 cutoff. We removed non-SNPs out of all variants and Tv out of SNPs from the consideration because these definitions gave identical R coefficient estimates as SNPs out of all variants and Ts out of SNPs, respectively. The *p* value, which has an inverse relationship with the t value, of the estimates of the R coefficients also can be used as a cutoff. Using *p*-value based cutoffs, the median R coefficient value is 9.8 with *p* < 0.05 (n = 23) and 9.2 with *p* < 0.01 (n = 17) (Fig 4A, S13 Table). Using Benjamini-Hochberg method [43], *p* ~ 0.08 for the R coefficient was considered significant using false discovery rate of 0.25 whereas *p* ~ 0.015 was considered significant using false discovery rate of 0.05. Using the variants that do not affect CDS or using all variants instead, the median estimates of the R coefficient change only slightly (S14 and S15 Tables, median R coefficient is 10.8 and 10.6, respectively, using *p* < 0.05 cutoff). Given these estimates and their accompanying supporting statistic values, it seems reasonable to propose an approximate R coefficient value of 10 for *C*. *elegans*. The estimates of F_M_ and F_S_ coefficients presuming a uniform R coefficient value (e.g. R = 10) are presented in S18 Table. An R coefficient of 10 suggests that 22% of all the variants in *C*. *elegans* are generated by the Morgan mechanism in the wild environment.

It is possible that the proposed R coefficient value of 10 is too big. With additional processing of overlapping variants and censoring of overshadowed variants (see Methods), the median R coefficient estimate is 5.5 using the variants that do not affect exons (*p* < 0.05 cutoff, S16 Table).

We reasoned that a range of acceptable R coefficient value also could be obtained by examining the quality of F_M_ and F_S_ coefficient estimates from LS regression analysis using a presumed the value of the R coefficient. By definition, the F_M_ and F_S_ coefficients must be in a range between 0 and 1. Thus, we declared that a presumed R coefficient value that suggests an F_M_ or F_S_ coefficient value outside this range is incorrect. We examined the estimates of the F_M_ and F_S_ coefficients using presumed R coefficient values between 0.01 and 9900. Here, an upper limit of 57 for the R coefficient is suggested by the analysis of indels of 1 base change (i4) out of non-SNPs whereas a lower limit of ~0.9 is suggested by i40-699 out of all variants (S4 Fig, S13 Table). By this criterion, 0.9 and 57 can be considered the lower and upper limits, respectively, of the R coefficient in *C*. *elegans*. This range is compatible with the proposed R coefficient value of 10 as well as the alternate R coefficient value of 5.5, which suggests a 34% contribution by the Morgan mechanism.

### Selection is likely responsible for smaller R coefficient estimates from variant distribution analysis

**Fig 5:**
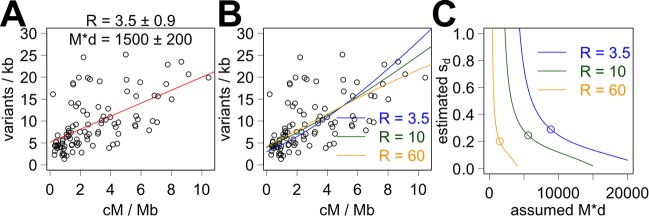
Fitting the correlation between the variant distribution and recombination rate without and with background selection. (A) Red line indicates the line of best fit using the mutation model alone. (B) Colored lines indicate the lines of best fit using combination of the mutation model with background selection and using three different presumed R coefficient values. (C) Colored lines indicate the estimates of s_d_ coefficients, and circles indicate the s_d_ estimate with the smallest *p* value. The circles in panel C indicate the values of s_d_ and M*d used for the lines of best fit in panel B. The presumed value of U is 0.48, and the presumed value of the outcrossing rate is 1.7%.

Variant distribution can be analyzed using the mutation model, and LS regression analysis should allow a different method of estimation of the R coefficient as well as estimation of the product of the Morgan coefficient and the divergence factor (M * d). Again, our standard analysis uses the variants that do not affect exons. Here, the estimate of the R coefficient is 3.5±0.9 (Fig 5A, S19 Table), and the estimate of M * d is 1500±200. This R coefficient estimate of 3.5 is accompanied by a large t value (t = 3.9) and a small *p* value (*p* < 0.0002), which indicate a higher degree of confidence than the R coefficient estimates derived from the analysis of variant composition. Using all variants and the variants that do not affect CDS instead, R coefficient estimates are 3.3±1 and 3.5±0.9 (S19 Table). The DNA length of the interval was adjusted when using a subset of variants by subtracting the length of DNA occupied by exons or CDS. Processing of overlapping variants and censoring of overshadowed variants were not performed for the analysis of variant distribution. Notably, this R coefficient estimate of 3.5 is considerably smaller than the proposed R coefficient value of 10 derived from the analysis of variant composition. R coefficients of 3.5 and 10 predict up to a 3.8-fold and 2-fold difference, respectively, in the variant distribution between the regions of high and low recombination (Fig 4B).

The discrepancy in the R coefficient estimates between the analysis of variant distribution and the analysis of variant composition can be reconciled. Earlier, we mentioned that linkage-based selection models could also explain the skewing of distribution of variants according to the recombination rate. This means that a proper analysis of the correlation between the recombination rate and variant distribution must account for both mutation and the Hill-Robertson effect in general (e.g. through background selection or selective sweep). On the other hand, the Hill-Robertson effect can be ignored in the analysis of variant composition. This means that an R coefficient of 10 from the analysis of variant composition reflects the action of mutation only whereas an R coefficient estimate of 3.5 from the analysis of variant distribution reflects the combined action of mutation and selection.

### Combined model of mutation and background selection

A more complete depiction of how the variant landscape is shaped is possible by combining our mutation model with a linkage-based selection model that also affects variant distribution according to the recombination rate. We chose background selection for a demonstration here because background selection is generally assumed to have a greater effect than selective sweep in *C*. *elegans*. A combined model containing both the background selection and our mutation model can be made by a coupling of the equations from the two models as follows:

$$v_{b}=\;\left(r\;+\;R\right)\;*\;d\;*\;n\;*\;M\;*\;\exp(\text{-}U\;/\;(s_{d}+\;r\;*\;\left(1\;\text{-}\;F\right)))\;/\;\left(1\;+\;F\right).$$

Here, mutation model equation v_o_ = (r + R) * d * n * M was used to substitute the number of variants without background selection (v_o_) in the background selection equation. Here, v_b_ is the number of variants after background selection, U is the deleterious mutation rate, s_d_ is the average selection coefficient, and F is the inbreeding coefficient, with a relationship to the outcrossing rate, c: F = (1-c)/(1+c) [4, 7]. Both s_d_ and F coefficients should have values between 0 and 1. Because background selection does not affect variant composition, the R coefficient estimate from the analysis of variant composition should be used here.

Others have determined some of the values used in the background selection model. The deleterious mutation rate (U) in *C*. *elegans* has been measured by many approaches with a wide range from 0.003 to 0.48, and 0.48 seems to be the current accepted value [44–49]. The rate of outcrossing for wild *C*. *elegans* has been estimated in a range between ~1% and 1.7% previously [50–52], except for an outlier estimate of 22% [53]. Thus, we performed LS regression analysis using presumed values of 0.48 and 1.7% for the U and outcrossing rate, respectively.

The estimates of the s_d_ selection coefficient vary depending on the assumed values of the R coefficient and M * d (Fig 5C). Here, our proposed R coefficient value of 10 was used alongside alternate values of 3.5 and 60. The estimates of s_d_ change depending on the assumed value of M * d, and the range of acceptable values of M * d also changes depending on the presumed R coefficient value. The estimates of s_d_ with the best t and *p* values were between 0.2 and 0.29 using presumed R coefficient values of 3.5, 10 and 60. These best s_d_ and M * d values were used along with the presumed R coefficient values to generate the lines of best fit between the recombination rate and the distribution of variants (Fig 5B). We suspect that bigger M * d values associated with larger presumed R coefficients simply reflect a bigger value of the M coefficient rather than a bigger divergence d value. Examining the sum of squares, the pseudo-R^2^ values are similar between the different presumed values of the R coefficient (R^2^ = 0.33 for R of 3.5, R^2^ = 0.43 for R of 10, R^2^ = 0.46 for R of 60). In comparison, R^2^ is 0.48 using the mutation model alone with the R coefficient of 3.5. Our opinion is that the differences in the R^2^ values are too small to be used to pick the value of the R coefficient. Nevertheless, these analyses using the combined model allow a fuller exploration of how variant landscape have been generated than by using a single model.

### The estimates of the R coefficient and significance

If mutation can generate a disparity in the landscape of variant composition according to the recombination rate, then natural selection theoretically can amplify or reduce the disparity by a simple mechanism. Take a scenario where two genomic regions accumulate different numbers of variants in the absence of selection (e.g. region A with 500 indels and 500 SNPs and region B with 20 indels and 80 SNPs). For simplicity, all variants are outside exons. Now, extend the scenario so that selection removes a higher percentage (e.g. 50%) of the indels and a lower percentage (e.g. 0%) of the SNPs. Here, selection has changed the variant composition (and distribution) to a different degree for the two regions, and the recombination rate is one of the underlying factors. In a simulation using the pf boundary definition, the real R coefficient value of 10, F_M_ for SNP of 0.8, and F_S_ for SNP of 0.7 without selection, introducing the action of selection that removes 50% of the indels and none of the SNPs would change the variant composition so that LS regression analysis would generate an apparent R coefficient estimate of 10.6 instead of 10. Selection is likely to remove indels more than SNPs and larger indels more than smaller indels, and F_M_ values are larger than F_S_ values for most indel types for *C*. *elegans*. Thus, selection in most cases would reduce rather than amplify the difference in the variant composition between the regions of low and high recombination. This means that our R coefficient estimates probably underestimate the real contribution of the Morgan mutation mechanism. We suspect that selection would discriminate between indels and SNPs to a lesser degree than in the example given here (i.e. 50% versus 0%), and thus the actual effect on the estimation of the R coefficient is probably smaller. As discussed earlier, DNA repair and apoptosis in direct response to mutation can also affect variant composition in a similar manner.

Further improvements in the estimation of the R and other coefficients may be possible through data aggregation using multiple interval boundary definitions (S12 Table). Here in addition to the standard pf boundary definition, we used three other definitions (pr for physical reverse, gf and gr for genetic forward and reverse, which are 5 cM-based intervals). The estimates of R coefficient from variant composition analysis fluctuate considerably with different interval boundary definitions (S13–S17 Tables). This effect of the genomic interval boundary choice is considerably larger than the effect of removing variants affecting exons or CDS from the analysis. For example using the variants that do not affect exons in the analysis of variant composition, the median R coefficient estimates are smaller using the other three interval boundary definitions (S13 Table, median R = 9.8, 9.1, 5.2, and 3.1 with a cutoff of *p* < 0.05 using pf, pr, gf, and gr interval boundary definitions, respectively). After aggregating the data using all four boundary definitions, the median R coefficient estimate is 7.2, which suggests a 32% contribution by the Morgan mechanism. It should be noted that the associated t and *p* values obtained using aggregated boundary definitions should be treated differently than when single boundary definition is used. Notably in the analysis of variant distribution, the R coefficient estimate also drops to 2.5 with the aggregated data using all four boundaries (S19 Table). Thus, a better R coefficient value may be 7 rather than 10, and a better estimate may be possible by more aggregation.

Errors in sequencing analysis, mutation detection, and mutation annotation are a potential problem in the analysis. More sequencing data, which is exemplified by a higher-quality sequencing data in the recently published genome of CB4856 [41], may help, but more important probably is a refinement in variant calling and better reconciliation of conflicting variant calls. Sequencing analysis can be problematic for highly repetitive sequences, which are enriched in the regions of high recombination. Homopolymer runs pose a different problem, and it is worth noting that Million Mutation Project (MMP) observed a more even distribution of mutations across the genome in artificially mutagenized *C*. *elegans* when homopolymer runs were excluded from the analysis [10]. Homopolymers presumably disproportionately affect variant calling of very small indels, and better estimates from LS regression analysis may be possible after accounting for homopolymers. Perhaps the most important factor is a large number of apparent conflicts in the variant annotation, which can be described as overlapping and overshadowed variants (see Methods). These conflicts appear to be a consequence of not reconciling the results of multiple independent mutation detection methods. From the analysis of variant composition using all four boundary definitions after accounting for these conflicts, we obtained a median R coefficient value of 4.8 (S16 Table), which suggests a 42% contribution by the Morgan mechanism. Other improvements in the analysis, such as a better calculation of the recombination rate of the interval when exons are removed from the analysis, may also be useful.

Our mutation model suggests that there are more variants in autosomal ends and in regions of high recombination in *C*. *elegans* in part because Morgan mechanism is a significant source of mutation generation. Perhaps Morgan mechanism plays a big part in mutation generation because double-strand breaks are inherently dangerous and liable to cause mutations. The dangers of breaking DNA, even when done deliberately, may also explain the higher proportion of larger indels associated with the Morgan mechanism. Alternatively, the fidelity of DNA replication may be more important for somatic cells than germ cells, and generating mutations in germ cells may facilitate evolution with minimal disruption in the development and physiology of individual organism. Similarly, larger indels may constitute a higher proportion of variants in the regions of high recombination in part because larger indels are more tolerated by DNA repair and apoptosis during meiosis.

A potential argument against our proposed R coefficient values between 4.8 and 10 comes from the analysis of mutation-accumulating (MA) strains [16, 17], which show a distribution of variants that cannot be reconciled with >2-fold difference between the regions of high and low recombination (Fig 4B). When the variant distribution in the MA strains is examined, the R coefficient estimate is 64±101 (*p* = 0.53) using the standard pf boundary definition and 35±18 (*p* = 0.05) using all four boundary definitions together. These R coefficient values suggest a 4% or 9% contribution by the Morgan mechanism, respectively. Meaningful analysis is not possible with variant composition in MA strains because the total number of MA variants is very small (n = 391). The authors of the MA strains suggested that the unequal distribution of variants in the wild population is likely a result of natural selection and not mutation. However, the generation of mutations in MA strains perhaps does not accurately reflect the generation of mutations in the wild environment, which is a possibility also suggested by the authors of the MA strain studies [16, 17]. Perhaps laboratory growth condition dramatically increases Sanger mechanism or decreases Morgan mechanism in *C*. *elegans*. If this is true, mutation-accumulating strains grown in laboratory may be irrelevant for studying the relative importance of Morgan and Sanger mutation mechanisms in the wild environment.

In this study, we showed that examining the composition of variants has a potential to reveal many interesting facets of molecular evolution. The proportions of many variant types in different genomic intervals show strong correlation with the recombination rate. By separating mutation mechanisms conceptually according to those dependent on genetic and physical distances, our mutation model describes how the composition of variant as well as the distribution of variants in the genome may become uneven throughout the *C*. *elegans* genome. We used this mutation model to systemically and holistically estimate the probabilities of generating specific mutation types by the putative Morgan and Sanger mechanisms in *C*. *elegans*. Since this mutation model is compatible with existing natural selection models, such as background selection, a more comprehensive analysis of genetic diversity is now possible. We think that this new metric of the proportion of variant types together with our mutation model have a potential to be generally useful in the analysis of genetic diversity in other species. It would be especially interesting to see if a substantial contribution of Morgan mechanisms in mutation generation is the rule rather than an exception when variant composition is examined in other species including in humans.

## Methods

### Computing

For computational processing and analysis, custom scripts were written and executed using R [54] and R Studio. The R package minpack.lm by Andrej-Nikolai Spiess and Katherine M. Mullen was used to perform LS regression analysis using Levenberg-Marquardt algorithm. The R package seqinr maintained by Simon Penel was used to determine GC content. The R scripts along with source and output files, which are needed to execute the scripts, are available at Dryad digital repository [55].

### Obtaining the *C*. *elegans* variant data and genomic features

The *C*. *elegans* variant data and genomic features were obtained using the newest version WS256 of WormBase (http://www.wormbase.org) [40]. Most of the data were obtained using the WormBase Genome Browser GBrowse (http://www.wormbase.org/tools/genome/gbrowse/c_elegans_PRJNA13758/). Specifically, the variants were obtained by using Polymorphisms track of GBrowse. Other GBrowse tracks used were: Curated Genes, Curated Genes (Protein-coding), Curated Genes (noncoding), RNAseq for expression level, Tandem and Inverted Repeats for repetitive sequences, and various Histone Modifications ChIP-Seq (H3K4me1, H3K36me1, H3K9me1, H3K27me1, and H3K27me3) for chromatin state. GFF3 files were obtained using GBrowse in all cases except WIG (signal) files were obtained for chromatin state. Curated Genes contain the details on genetic and physical location as well as exons and introns, and Curated Genes (protein-coding) and Curated Genes (noncoding) contain the information specific to coding DNA sequences and non-coding RNA, respectively. ChIP-seq and RNAseq data were generated by the modENCODE project [56]. The WS256 version PRJNA13758 of the *C*. *elegans* genomic DNA sequence [27] was obtained using the WormBase FTP site (ftp://ftp.wormbase.org/pub/wormbase/species/c_elegans/sequence/genomic/). To obtain a list of essential genes, we used the queries lethal, sterile, larval lethal, larval arrest, embryonic lethal, and zygotic lethal as mutant phenotypes using WormMine version WS253 (http://intermine.wormbase.org/tools/wormmine/begin.do).

### Genomic intervals

We use four sets of interval boundary definitions (S12 Table). Two sets are organized by one megabase (Mb), and two other sets are organized by five centimorgans (cM). The boundaries are set starting from either the left or the right telomere of the chromosome. The final intervals at the chromosomal ends are less than 1 Mb or 5 cM of DNA, except any interval of smaller than 0.2 Mb is joined with a neighboring interval to minimize noise. The intervals from the different boundary definitions do not share common end points except for the telomeres. Total of 102 and 58 intervals exist according to the boundary definitions using 1 Mb and 5 cM, respectively.

### Exon, Intron, and repetitive sequence density

The total exon space was calculated after accounting for duplicates and overlaps. Specifically, duplicate exons that share the same 5' and 3' ends were removed. Overlapping exons were combined into one exon. Any exon that is located completely within another exon was removed. The same process was used to calculate the genomic space occupied by essential genes, the genomic space occupied by the exons of essential genes, and the genomic space occupied by repetitive sequences. For introns, an additional process of subtracting the space occupied by exons within introns was performed. The density of these elements is defined as the physical length of space occupied by the element (e.g. exons) divided by the physical length of the genomic interval.

### Expression level and chromatin state

RNAseq GFF3 file was used to calculate expression level, and WIG files were used to measure chromatin state. Briefly, we multiplied the interpreted depth of coverage by RNAseq by the length of each RNAseq reads. To obtain normalized expression level for each genomic interval, we then divided the sum of these products by the total length of the genomic interval. Similarly, the sum of the levels of methylation state for 10 base pair stretches in WIG files were obtained for each genomic interval. To obtain normalized chromatin state level for each genomic interval, we divided this sum by the number of the 10-base pair stretches corresponding to each genomic interval.

### Processing the variant data

The variant data obtained from WormBase using the Polymorphisms track of GBrowse contain a great deal of information. The information includes the start and end positions of the variants relative to the N2 reference genome. Other information includes a list of wild isolates with the variant and the consequence of the variant on the protein coding sequence.

We focused solely on 40 wild isolates out of hundreds of *C*. *elegans* wild isolates with variant data. The reason for choosing these 40 isolates was that these 40 have been sequenced via whole-genome sequencing by the Million Mutation Project (MMP) and thus have the most complete data. The 869,019 variants in the 40 wild isolates account for over 97% of all natural variant records in the WS256 WormBase polymorphism data. Four different methods were used by the MMP to identify variants [10], specifically a process using the standard suite of SAMtools software (mpileup, bcftools, vcfutils.pl), a two-step process involving scanning of mpileup for "gapped" reads to identify small indels (<200 bp), a process involving examination of "split" reads generated by phaster to identify indels of 100–5000 bp, and a process of examination of variations or changes in read coverage to identify larger copy number variants. The MMP-specific variant dataset is available separately (http://genome.sfu.ca/mmp/), but the physical position of the variants in the MMP curation often does not perfectly match the reference genome position provided by WormBase. Many of the variants annotated by WormBase were identified over a long period of time for some of the 40 wild isolates. Other methods of variant identification included light shotgun sequencing [21, 57] and oligo array comparative genome hybridization [22, 58, 59]. The WormBase GFF3 annotation includes the laboratory source of the variant data, which is only useful for distinguishing the variants from light shotgun sequencing from all other methods because the method of variant identification is not specified for each variant.

We found many inconsistencies of annotation in the WormBase curated variants, including adjacent, overlapping, and overshadowed variants. For example, substitutions of two or more adjacent base pairs are often not curated as a single variant even in cases of the adjacent variants being present in an identical set of wild isolates. Combining such adjacent variants seems absolutely warranted. More complicated are a small number of variants occupying an overlapping space in the genome. Arguably the most consequential are a large number of variants that are completely enveloped, or overshadowed, by a large deletion. Overlapping and overshadowing of variants may stem from complex genome rearrangement, such as a combination of deletion(s) and duplication(s). Another possible source of inconsistency is the differences in sample sources, but we found many similar inconsistencies using only the MMP data. Therefore, the main problem appears to be different results derived from multiple independent variant calling methods and a lack of a thorough and holistic reconciliation of the conflicting results.

After combining all adjacent variants that are present in identical set of wild isolates, the total number of variants was reduced from 869,019 to 853,815. Our main analysis was performed using these 853,815 processed variants either as a whole or as an aggregation in 37 of the 40 wild isolates. For further processing involving overlapping and overshadowed variants, we did not require that the variants of interest are present in identical set of wild isolates. Overlapping and overshadowing variants were processed at the level of individual wild isolates only, and these more processed variants were used for supplementary analysis. Overlapping variants were combined into a single variant, and overshadowed variants were censored. In part because of the presence of some very large indels including 144 variants of >100,000 base pair deletions (including a deletion of >1.4 Mb), this process reduced the number of variants by 11% to 28% depending on the wild isolate (median = 19%).

### Annotating variant data

The location of the variants was annotated relative to exons, introns, CDS, ncRNA, and repetitive sequences. The results of this annotation at the whole-genome level are summarized in S11 Table. Variants are counted as affecting a genetic feature (e.g. exon) if at least one base pair of the genetic feature was changed. Variants were counted as being inside a genetic feature only if no DNA outside the genetic feature was affected. Similarly, variants were counted to be within an interval only if both the starting position and the end position of the variant lie within the defined interval. By our definition, the variants that do not affect exons are the variants that do not affect any of the following genetic features: coding DNA sequence (CDS), 5' UTR, 3' UTR, pseudogene, and non-coding RNA (i.e. miRNA, tRNA, rRNA, snRNA, snoRNA, piRNA, lincRNA, scRNA). CDS is the sole consideration for annotation of the variants that do not affect CDS.

WormBase annotations were used to check our annotation results that were examined relative to CDS (n = 146,746). All but 70 variants that we annotated as affecting CDS had WormBase annotation term = Nonsense, = Frameshift, = Silent, = Coding_exon, or = Readthrough. In the case of these 70 exceptions, WormBase used the annotation term = Splice_site instead. The conflict is there because WormBase used a shorter exon rather than a larger exon in these 70 cases. Conversely, our annotation of the variants that affect CDS missed 26 variants that WormBase annotated with the terms = Frameshift or = Coding_exon. Of these 26, WormBase correctly annotated 14 insertions affecting splice site as = Frameshift. WormBase annotation looked incorrect to us in the other 12 cases.

## Supporting Information

S1 Text Worksheet for the equations of the mutation model. (DOCX)

S1 Table The composition of variants and the recombination rate. Relationship between the recombination rate and the proportion of variant types were examined by correlation tests and linear regression analysis. Total of 58 definitions of the variant type proportion were examined.
(CSV)

S2 Table The distribution of variants and the recombination rate. Relationship between the recombination rate and the distributions of variants were examined by correlation tests and linear regression analysis. Total of 31 variant types were examined.
(CSV)

S3 Table The composition of variants and other genomic features. Relationship between the proportion of variant types and other genomic features were examined by correlation tests and linear regression analysis. Other genomic features are exon density, CDS density, essential gene density, essential gene exon density, repetitive sequence density, GC content, RNAseq level, and chromatin state.
(CSV)

S4 Table The distribution of variants and other genomic features. Relationship between the distribution of variants and other genomic features were examined by correlation tests and linear regression analysis.
(CSV)

S5 Table The composition of variants and the recombination rate for the variants that do not affect exons. Relationship between the recombination rate and the proportion of variant types were examined by correlation tests and linear regression analysis using the variants that do not affect exons.
(CSV)

S6 Table The composition of variants and the recombination rate for the variants that affect exons. Relationship between the recombination rate and the proportion of variant types were examined by correlation tests and linear regression analysis using the variants that affect exons.
(CSV)

S7 Table The composition of variants and the recombination rate for the variants that affect repetitive sequences. Relationship between the recombination rate and the proportion of variant types were examined by correlation tests and linear regression analysis using the variants that affect repetitive sequences.
(CSV)

S8 Table The composition of variants and the recombination rate for the variants that do not affect repetitive sequences. Relationship between the recombination rate and the proportion of variant types were examined by correlation tests and linear regression analysis using the variants that do not affect repetitive sequences.
(CSV)

S9 Table The composition of variants and the recombination rate for the variants that affect the DNA outside repetitive sequences. Relationship between the recombination rate and the proportion of variant types were examined by correlation tests and linear regression analysis using the variants that affect the DNA outside repetitive sequences.
(CSV)

S10 Table The composition of variants and the recombination rate for the variants that only affect the DNA inside repetitive sequences. Relationship between the recombination rate and the proportion of variant types were examined by correlation tests and linear regression analysis using the variants that only affect the DNA inside repetitive sequences.
(CSV)

S11 Table The number of variants in the 40 wild isolates of *C. elegans*. Breakdown of the total number of variant types are given for all variants as well as for the variants that affect or do not affect exons, CDS, repetitive sequences and other features.
(CSV)

S12 Table Genomic interval boundaries and characteristics. The four boundary definitions (pf, pr, gf, gr) and their characteristics are described.
(CSV)

S13 Table Coefficients derived from LS regression analysis using the variants that do not affect exons. The results of analysis are shown using the aggregated data from 37 wild isolates.
(CSV)

S14 Table Coefficients derived from LS regression analysis using the variants that do not affect CDS. The results of analysis are shown using the aggregated data from 37 wild isolates.
(CSV)

S15 Table Coefficients derived from LS regression analysis using all variants. The results of analysis are shown using the aggregated data from 37 wild isolates.
(CSV)

S16 Table Coefficients derived from LS regression analysis after additional variant processing. Overlapping variants were combined, and overshadowed variants were censored. The variants that affect exons were censored. The results of analysis are shown using the aggregated data from 37 wild isolates.
(CSV)

S17 Table Coefficients derived from LS regression analysis without data aggregation. The results of analysis are shown using the variants that do not affect exons without data aggregation.
(CSV)

S18 Table Estimates of FM and FS coefficients using presumed values of the R coefficient. Presumed R coefficient values are 6, 10 and 60.
(CSV)

S19 Table Coefficients derived from LS regression analysis of the distribution of variants. The results of analysis are shown using all variants, the variants that do not affect exons, and the variants that do not affect CDS.
(CSV)

S1 Fig Chromosomal patterns of the characteristics of genomic intervals. Chromosomal patterns are shown for the proportion of i40-699 out of all variants (A), proportion of SNPs out of all variants (B), recombination rate (C), exon density (D), intron density (E), repetitive sequence density (F), GC content (G), and expression level (H).
(TIF)

S2 Fig Correlations with the recombination rate for different chromosomes. Correlations are shown for the proportion of i40-699 out of all variants (A), the proportion of SNPs out of all variants (B), the proportion of i40-699 out of non-SNPs (C), the proportion of transitions (Ts) out of SNPs (D), and the distribution of all variants (E).
(TIF)

S3 Fig Correlations with the recombination rate for subpopulations of variants. Analysis using the variants that affect exons, the variants that do not affect exons, the variants that affect introns but not exons, the variants that affect neither CDS nor introns, the variants that affect repetitive sequences, the variants that do not affect repetitive sequences, the variants that are located inside repetitive sequences, and the variants that are not inside repetitive sequences. Correlations are shown for the proportion of i40-699 out of all variants (A), the proportion of SNPs out of all variants (B), the proportion of i40-699 out of non-SNPs (C), and the proportion of transitions (Ts) out of SNPs (D).
(TIF)

S4 Fig Acceptable range of presumed R coefficient values according to the estimates of FM and FS. Estimates of F_M_ (black) and F_S_ (red) obtained with presumed R coefficient value in the x-axis while examining the correlation between various variant type proportions and the recombination rate. Vertical hatched lines indicate the lower and upper limits of acceptable R coefficient values. The lower limit of the acceptable R coefficient was not determined for i1/variants, i4/variants, and i4/non-SNPs.
(TIF)
