# Supplementary material for: Effect of mutation mechanisms on variant composition and distribution in Caenorhabditis elegans
Source: PLoS Comput Biol. 2017 Jan 30;13(1):e1005369. doi: 10.1371/journal.pcbi.1005369 (PMC5305269; doi:10.1371/journal.pcbi.1005369)
Supplement: S1 Text — (DOCX) [file pcbi.1005369.s001.docx]

**Supplementary Text 1. Worksheet for the equations of the mutation model.**

Morgan mechanism produces variants according to the genetic distance of the interval whereas Sanger mechanism produces variants according to the physical distance of the interval. A key assumption is that all mutation rates over evolutionary time stay constant. The properties of the Morgan and Sanger mechanisms in mutation generation are summarized below.


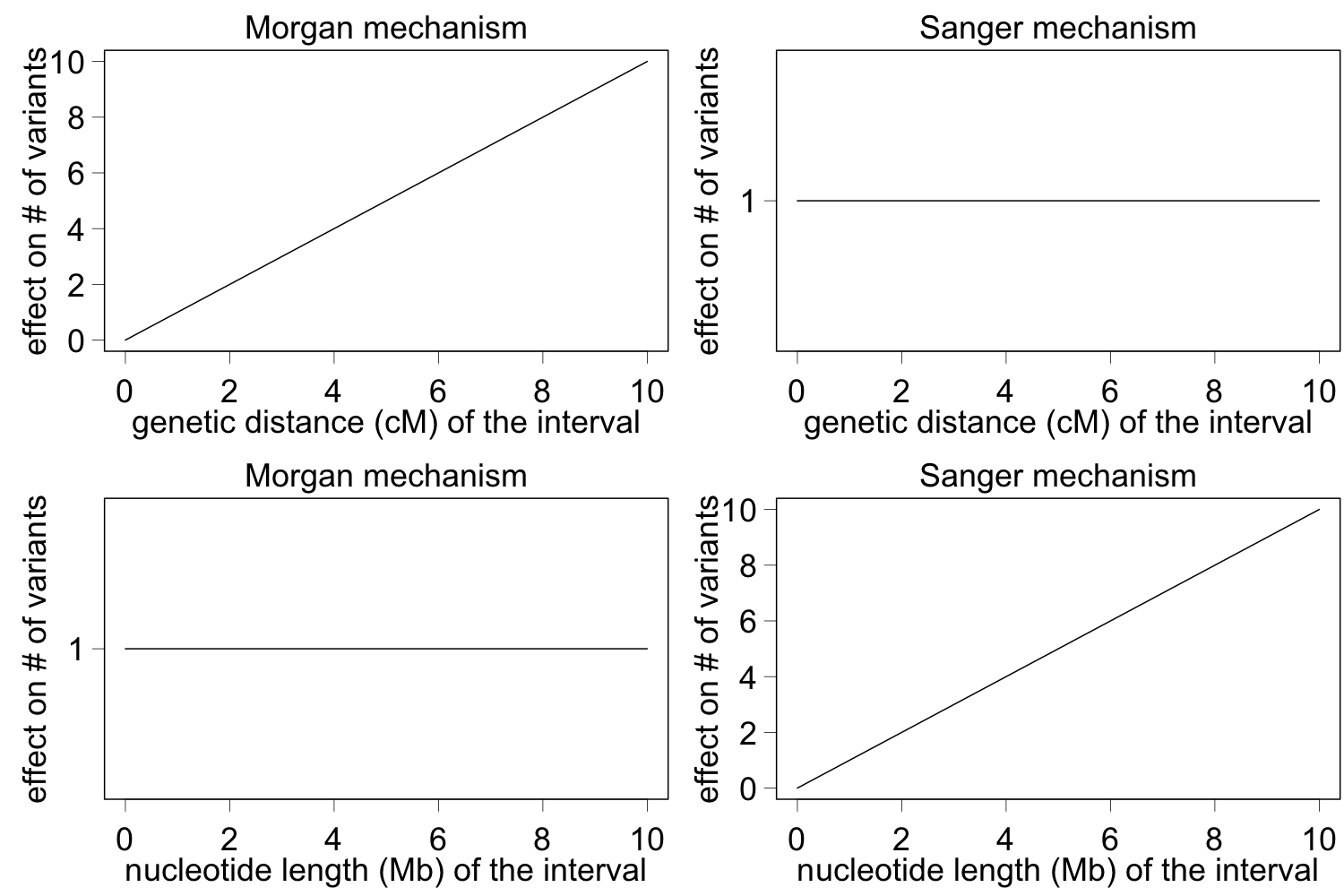


For an interval of g cM and Morgan coefficient (a constant):

# of variants by Morgan mechanism = Morgan coefficient [cM^-1^] * g [cM]

For an interval of n Mb and Sanger coefficient (another constant):

# of variants by Sanger mechanism = Sanger coefficient [Mb^-1^] * n [Mb]

A fixed ratio that we call the R coefficient describes the relationship between the Morgan and Sanger coefficients:

R coefficient [cM/Mb] = Sanger coefficient [Mb^-1^] / Morgan coefficient [cM^-1^]

Sanger coefficient [Mb^-1^] = R coefficient [cM/Mb] * Morgan coefficient [cM^-1^]

Now, the number of variants by the Morgan and Sanger mechanisms can be rewritten as follows:

# variants by Morgan = g [cM] * Morgan coefficient [cM^-1^]

= g [cM] / n [Mb] * n [Mb] * Morgan coefficient [cM^-1^]

= r [cM/Mb] * n [Mb] * Morgan coefficient [cM^-1^]

# variants by Sanger = n [Mb] * Sanger coefficient [Mb^-1^]

= n [Mb] * R coefficient [cM/Mb] * Morgan coefficient [cM^-1^]

For ease of read, we will use the following shorthands. The recombination rate of the interval (g cM / n Mb) is r; the physical length of the interval (n Mb) is n; the Morgan coefficient is M in units of cM^-1^; and the R coefficient in units of cM/Mb is R. While we used cM and Mb as units, another unit, such as kb, can be used instead. More importantly, the Morgan and Sanger mechanisms need to be normalized to represent the same time scale. A generation is suitable for normalization, and we use symbol d for divergence to represent generations. The number of all variants v_o_ in a given interval per generation is:

v_o_ = r [cM/Mb] * n [Mb] * M [cM^-1^] * d + R [cM/Mb] * n [Mb] * M [cM^-1^] * d

= (r + R) * n * M * d

~ µ for the entire genome using sum of r, sum of n, and R * # of all intervals

All variants generated by the Morgan mechanism are r * n * M * d, and all variants generated by the Sanger mechanism are R * n * M * d after normalization using d. It is probably reasonable to think of v_o_ at the whole-genome level as being equivalent to the mutation rate µ, which is commonly used in population genetics.

By combining all expected contributions in all intervals, the basic equation above can be used to determine the expected contributions of the Morgan and Sanger mechanisms throughout the whole genome as written below with sum of R being equal to R * total number of intervals.

overall proportion by Morgan for the whole genome = Σ r / (Σ R + Σ r)

overall proportion by Sanger for the whole genome = Σ R / (Σ R + Σ r)

Assuming that each mutation mechanism generates specific mutation types with a constant probability, the number of a specific variant type s_o_ is:

s_o_ = F_M_ * r * n * M * d + F_S_ * R * n * M * d

= (F_M_ * r + F_S_ * R) * n * M * d

Here, s_o_ is the total number of a specific mutation type generated at a given interval. Two coefficients, F_M_ and F_S,_ are the probability of generating that specific mutation type by the Morgan and Sanger mechanisms, respectively. The F_M_ and F_S_ coefficients are applicable to the whole genome for a given species. While coefficients F_M_, F_S,_ and R are assumed to be stable throughout the evolutionary history of a particular species, we note that a variety of factors can potentially cause changes in these values. For example, changes in environment, such as an increase in UV irradiation, should affect both the R and F_S_ coefficients, resulting in an increased role of non-Morgan mutation and probably more indels as a result of random DNA breaks. Similarly, certain mutations, such as mutations in the repair pathway for double-strand DNA breaks, can affect both the R and F_M_ coefficients. However, once again we have assumed, for simplicity, that these factors stay essentially constant in our analysis.

Some of the equations introduced thus far can be combined to predict the percentage of a specific mutation type at a given interval, as follows:

f_o_ = s_o_ / v_o_

= ((F_M_ * r + F_S_ * R) * n * d * M) / ((r + R) * n * d * M)

= (F_M_ * r + F_S_ * R) / (r + R)

A convenient part of this equation for the proportion of a specific mutation type (f_o_) is that divergence does not matter as long as the mode of mutation stays constant throughout the evolutionary history of a given species. The Morgan coefficient M also does not matter as it is cancelled out. Selective sweeps and background selection should have no effect on the f_o_.

The same equation can be used to predict the percentage of a specific variant type within a larger group of variant types; for example, i40699 out of non-SNPs with v_o_ here used to describe the density of non-SNPs rather than the density of all variants:

s_o1_ / s_o2_ = ((F_M1_ * r + F_S1_ * R) * n * d) / ((F_M2_ * r + F_S2_ * R) * n * d)

= (F_M1_ * r + F_S1_ * R) / (F_M2_ * r + F_S2_ * R)

= (F_M1_ / F_M2 *_ r + F_S1_ / F_S2_ * R) / (r + R)

= (F_M_ * r + F_S_ * R) / (r + R)

Here, F_M1_/F_M2_ and F_S1_/F_S2_ are the probability of generating a variant subtype 1 out of a variant subtype 2, which includes the variant subtype 1. These coefficients are constants like F_M_ and F_S_, and they can be referred to as F_M_ and F_S_ as long as it is clear that a different denominator other than all variants are being used.
