# Supplementary figures and images for: Effect of mutation mechanisms on variant composition and distribution in Caenorhabditis elegans
Source: PLoS Comput Biol. 2017 Jan 30;13(1):e1005369. doi: 10.1371/journal.pcbi.1005369 (PMC5305269; doi:10.1371/journal.pcbi.1005369)

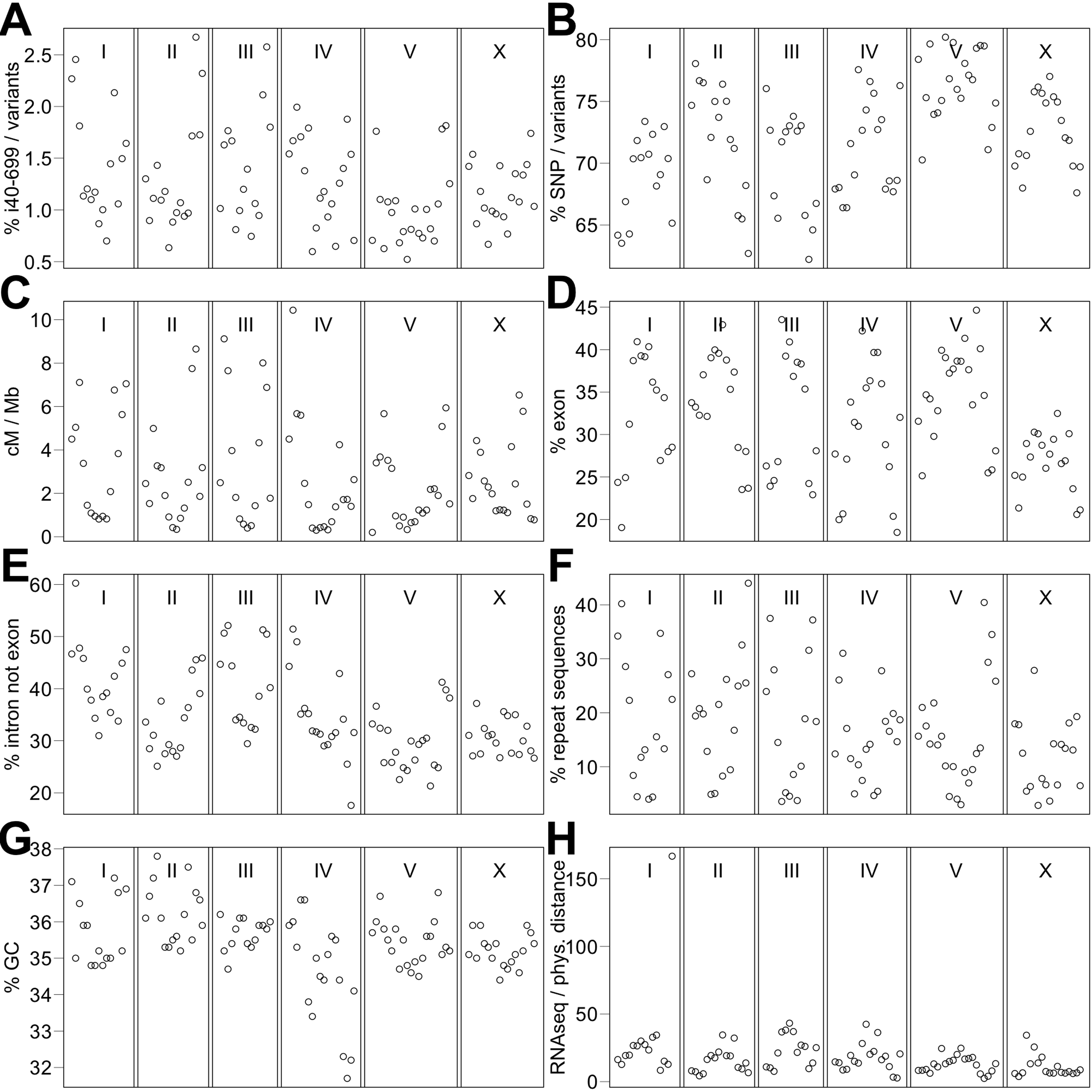

Supplement: S1 Fig — Chromosomal patterns are shown for the proportion of i40-699 out of all variants (A), proportion of SNPs out of all variants (B), recombination rate (C), exon density (D), intron density (E), repetitive sequence density (F), GC content (G), and expression level (H). (TIF) [file pcbi.1005369.s021.tif]

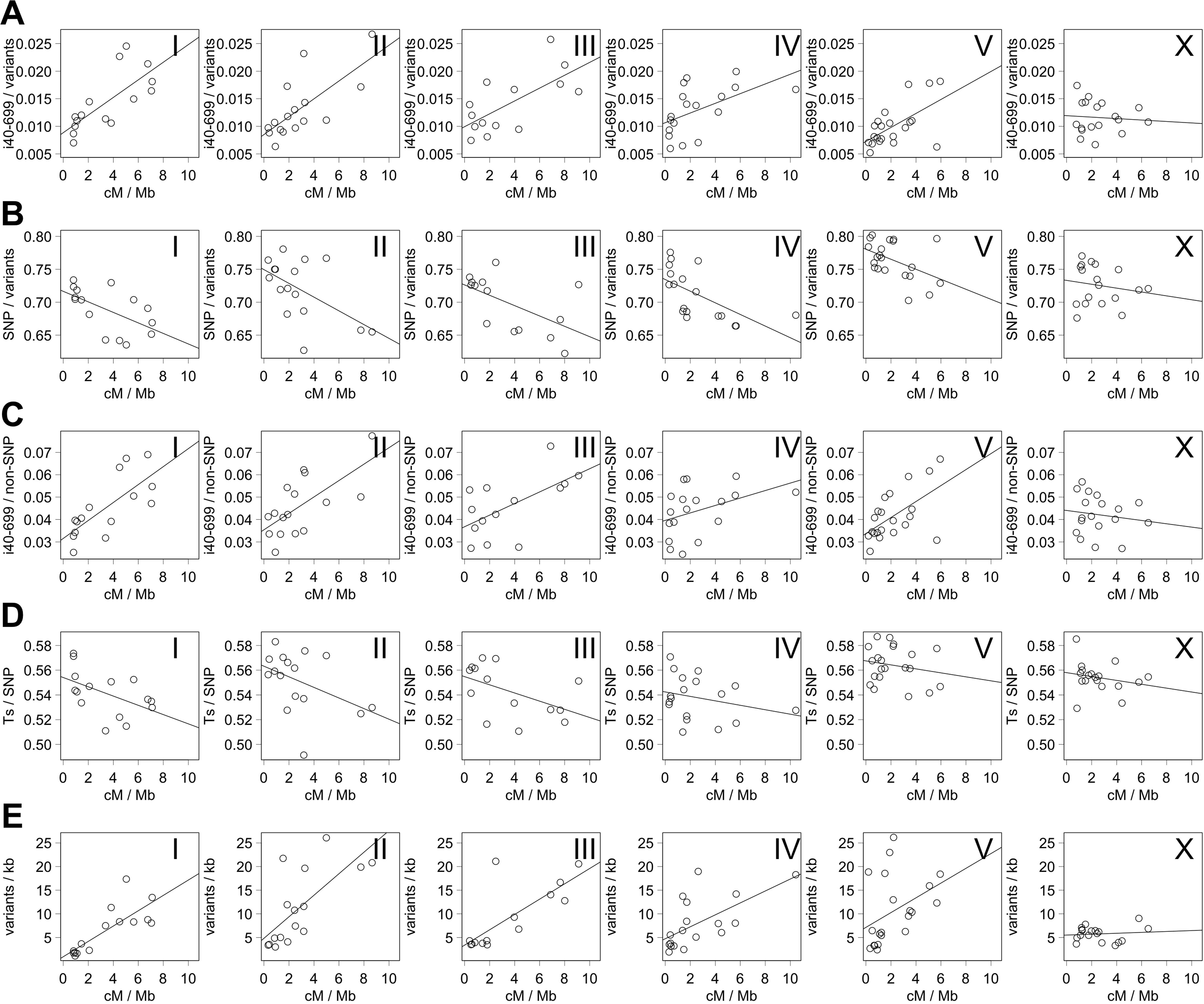

Supplement: S2 Fig — Correlations are shown for the proportion of i40-699 out of all variants (A), the proportion of SNPs out of all variants (B), the proportion of i40-699 out of non-SNPs (C), the proportion of transitions (Ts) out of SNPs (D), and the distribution of all variants (E). (TIF) [file pcbi.1005369.s022.tif]

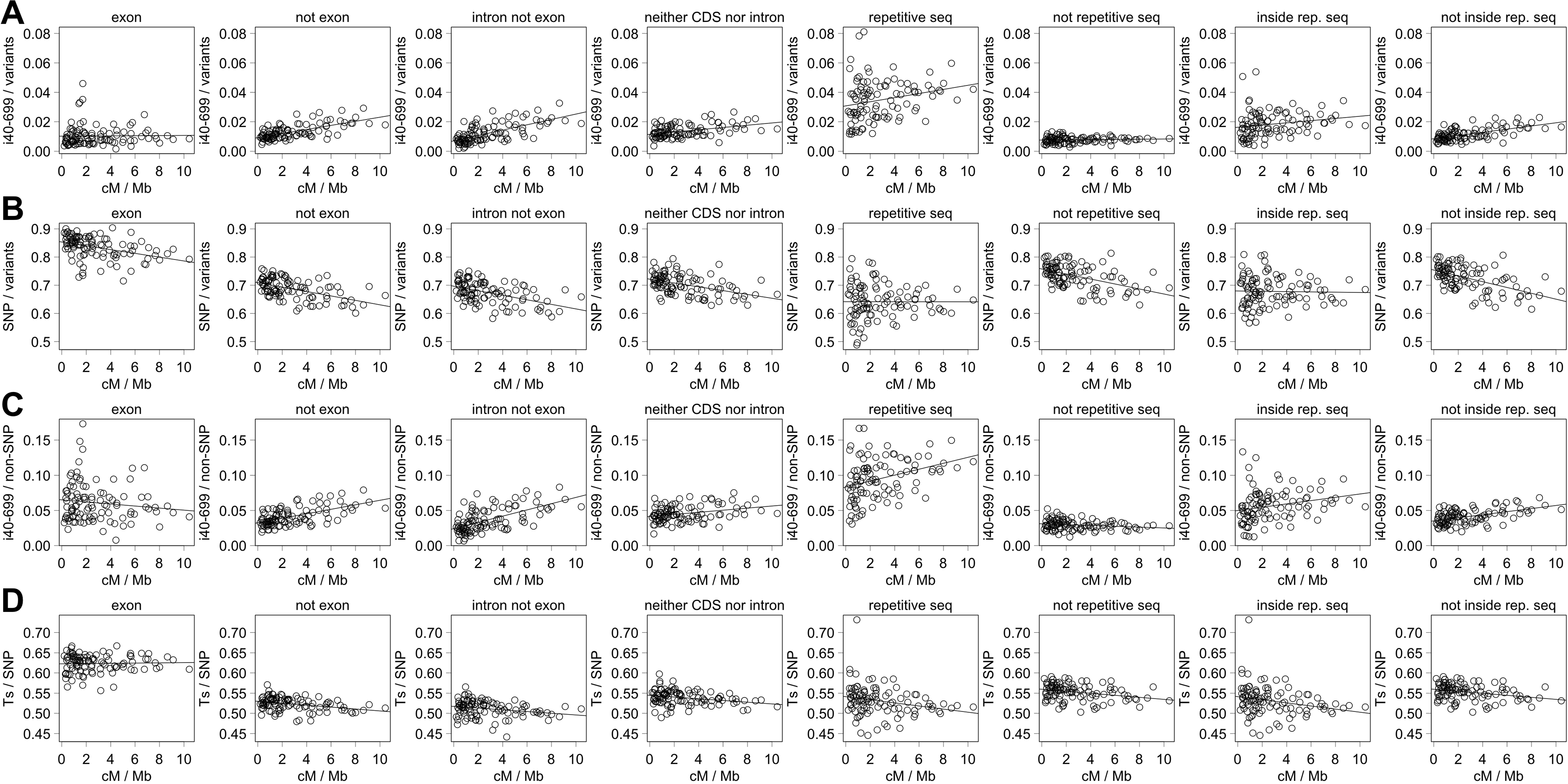

Supplement: S3 Fig — Analysis using the variants that affect exons, the variants that do not affect exons, the variants that affect introns but not exons, the variants that affect neither CDS nor introns, the variants that affect repetitive sequences, the variants that do not affect repetitive sequences, the variants that are located inside repetitive sequences, and the variants that are not inside repetitive sequences. Correlations are shown for the proportion of i40-699 out of all variants (A), the proportion of SNPs out of all variants (B), the proportion of i40-699 out of non-SNPs (C), and the proportion of transitions (Ts) out of SNPs (D). (TIF) [file pcbi.1005369.s023.tif]

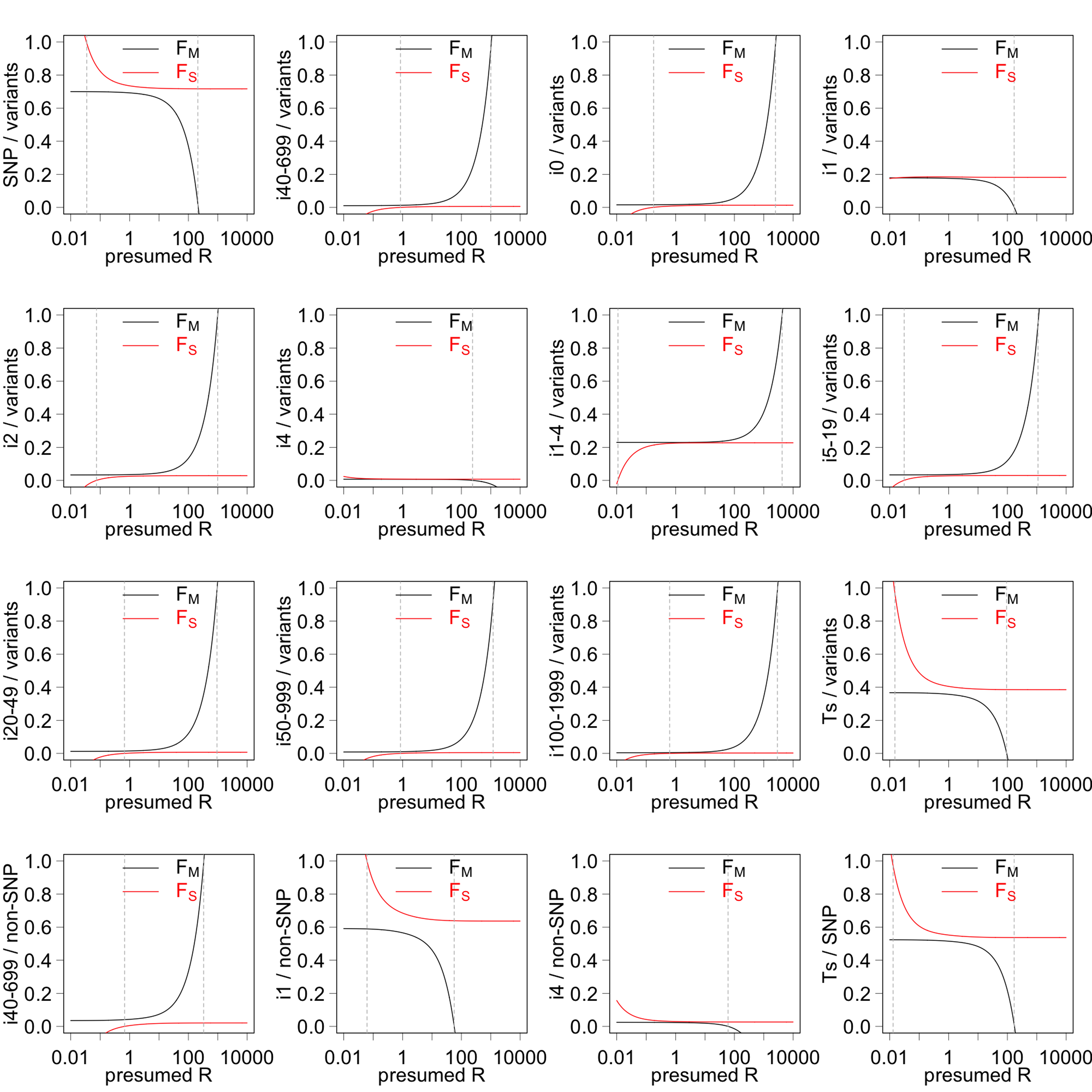

Supplement: S4 Fig — Estimates of FM (black) and FS (red) obtained with presumed R coefficient value in the x-axis while examining the correlation between various variant type proportions and the recombination rate. Vertical hatched lines indicate the lower and upper limits of acceptable R coefficient values. The lower limit of the acceptable R coefficient was not determined for i1/variants, i4/variants, and i4/non-SNPs. (TIF) [file pcbi.1005369.s024.tif]
